# Research advances in nanomaterials with aromatic functional groups for the treatment of inflammatory bowel disease

**DOI:** 10.3389/fbioe.2025.1614939

**Published:** 2025-06-24

**Authors:** Juan He, Hong Guo, Min Zou

**Affiliations:** ^1^ Chongqing Medical University, Chongqing, China; ^2^ Chongqing General Hospital, Chongqing, China

**Keywords:** IBD, nanomaterials, aromatic functional groups, inflammatory, treatment

## Abstract

Inflammatory bowel disease (IBD) is a group of chronic, non-specific intestinal inflammatory diseases. The treatment of IBD focuses on alleviating intestinal inflammation. However, traditional drug treatment methods are limited by the side effects of systemic therapy, poor targeting of oral administration, and low bioavailability at the site of inflammation. Fortunately, the application of nanomaterials in the intestine is expected to alleviate these problems. Nanomaterials have unique physicochemical properties and can be used for targeted drug delivery through the mucus layer. Moreover, they can also be engulfed by macrophages through endocytosis, thereby regulating the immune environment of the intestine and potentially altering drug properties. In addition, nanomaterials can be divided into organic nanomaterials and inorganic nanomaterials according to their properties. The functional groups in organic nanomaterials directly determine the characteristics and effects of these materials. Among them, aromatic rings enhance drug stability, improve drug solubility and targeting, and exert anti-inflammatory, antioxidant, and immunomodulatory effects, which are conducive to the innovation of IBD treatment. This paper focuses on the role of aromatic rings, briefly describes the current therapeutic status of organic nanomaterials in inflammatory bowel disease, and discusses the deficiencies of existing research as well as directions for future studies. This paper provides insights into understanding the influence of functional groups on nanomaterials.

## 1 Introduction

IBD is a group of chronic non-specific inflammatory diseases of the intestine, encompassing Ulcerative colitis and Crohn’s disease. Despite extensive efforts to elucidate the pathophysiological mechanisms underlying the onset of IBD, the exact etiology remains incompletely understood. To date, several factors have been implicated as potential causative agents, including genetic predisposition, immunoregulatory disorders, and microbiome dysregulation, all of which are regarded as critical contributors to the development of IBD. Over the past decade, the marked increase in IBD prevalence has placed a significant financial strain on global public healthcare systems (Kaplan, 2015; Kaplan and Windsor, 2021; Kumar et al., 2023). Conventional non-targeted pharmacological agents frequently employed in the clinical management of IBD, including 5-aminosalicylic acid (5-ASA), glucocorticoids, and immunosuppressants, are known to exert anti-inflammatory effects through the reduction of inflammatory mediator synthesis or the modulation of immune system activity (Dubois-Camacho et al., 2017). However, this systemic administration method results in poor drug targeting, requiring a high dosage to achieve the effective drug concentration. This increases the likelihood of adverse reactions in patients, such as nausea, vomiting, liver, and kidney toxicity, significantly limiting the clinical application of these drugs. Over the past 20 years, various biological agents have emerged, such as tumor necrosis factor-α (TNF-α) inhibitors, interleukin-12/23 antibodies, and anti-α4β7 integrin antibodies (Aggeletopoulou et al., 2018; Rawla et al., 2018; Jang et al., 2021). These biological agents have shown clear efficacy in both inducing and sustaining clinical remission, as well as in facilitating mucosal healing. However, the prevalence of primary or secondary non-response remains relatively high, primarily due to issues related to immunogenicity (Gaujoux et al., 2019). Moreover, the administration methods of long-term intravenous infusion and subcutaneous injection lead to poor patient compliance. Over the last decade, numerous researchers have capitalized on the swift technological progress in nanomedicine to develop innovative Drug Delivery Systems (DDS). These DDS are designed with the objective of increasing the drug concentration in the targeted area by regulating the release of the Active Pharmaceutical Ingredient (API) or implementing cell-specific targeting approaches (Xin Li et al., 2020).

Aromatic functional groups constitute a category of functional groups characterized by aromaticity, which imparts distinct chemical properties and stability, rendering them particularly valuable in the fields of pharmaceuticals and materials science. Notable bioactive compounds containing aromatic functional groups include curcumin, proanthocyanidins, baicalein, and quercetin. The integration of these bioactive compounds with nanomaterials has the potential to augment their therapeutic efficacy in the management of IBD.

### 1.1 Methodology

**FIGURE 1:**
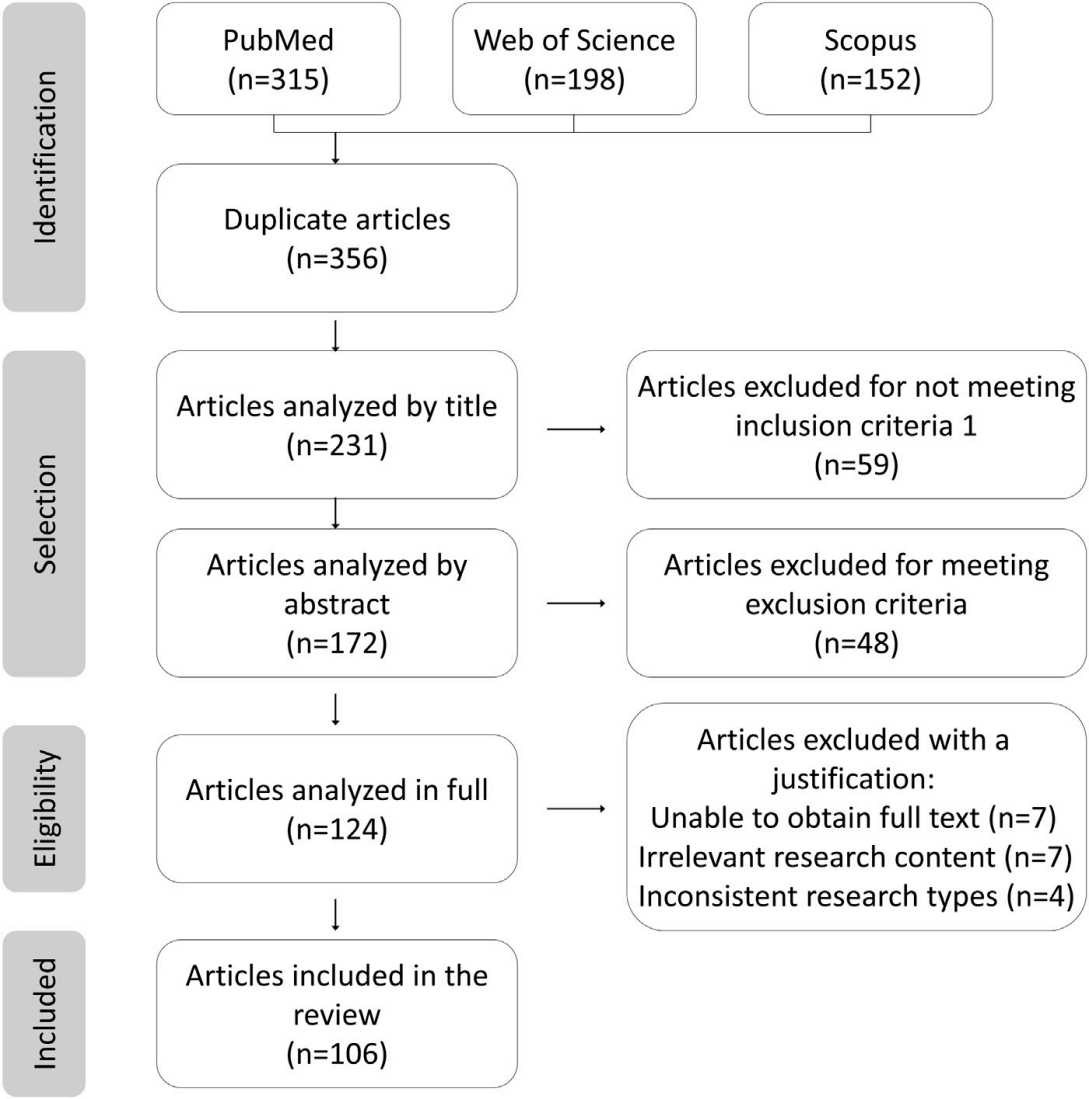
PRISMA flowchart.

The literature screening methodology followed PRISMA (Figure 1), detailing the databases used including PubMed, Web of Science, and Scopus, along with keywords “nanoparticles AND aromatic groups” and “inflammation AND nanocarriers” spanning 2005–2025. Studies included were preclinical/clinical research on aromatic‐functionalized nanomaterials and mechanistic analyses of biochemical interactions, while exclusions comprised non‐aromatic nanomaterials and purely computational studies without experimental validation.

## 2 Definition and physicochemical properties of aromatic functional groups

Aromaticity refers to the special stability and chemical properties exhibited by molecules with a conjugated ring system that satisfy Hückel’s rule. This rule requires molecules to have a planar, continuously conjugated ring structure with a π-electron count of 4n + 2 (where n is an integer, e.g., 0, 1, 2, 3, etc.) (Liu et al., 2019). Owing to their aromatic nature, these functional groups exhibit considerable stability in chemical reactions and are predisposed to undergo electrophilic substitution rather than addition reactions (Yu et al., 2021). The distinctiveness of aromatic functional groups is attributed to their π-conjugated electronic systems, which facilitate the formation of unique supramolecular structures through various mechanisms, including conjugated plane formation, nonlinear optical behavior, asymmetric modification, chirality, excited-state optimization, and directed assembly (Rimmele et al., 2021). Nitrogen atoms in graphite-like carbon nitride (g-C_3_N_4_) possess a high electron cloud density, which contributes to their exceptional performance in photocatalysis and fluorescence sensing. Regions characterized by high electron cloud density generally demonstrate increased reactivity. Consequently, aromatic functional groups, with their elevated electron cloud density, are more inclined to engage in redox reactions (Morales-Roque et al., 2009; Raúl Flores, 2016). During inflammatory processes, aromatic functional groups can mitigate the detrimental effects of free radicals and reactive oxygen species (ROS) through electron transfer reactions, thereby alleviating oxidative stress-induced tissue damage and exerting anti-inflammatory effects (Chakraborty et al., 2024). Additionally, the hydrophobic nature of aromatic functional groups allows nanomaterials containing these groups to more easily penetrate cell membranes and enter cells.

## 3 Nanomaterials: definitions, characterization, and applications in biomedicine

**FIGURE 2:**
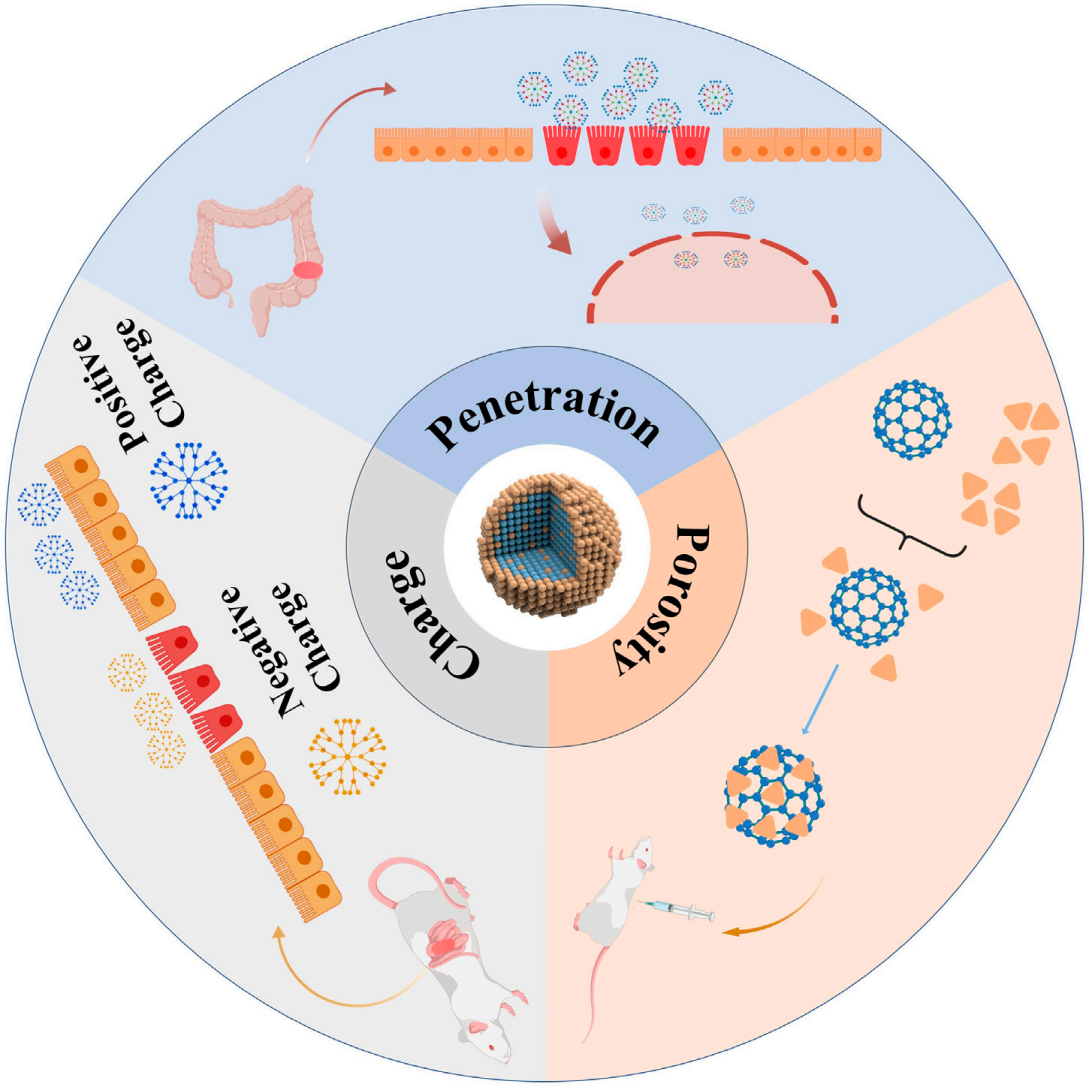
The characteristics exhibited by nanomaterials during the treatment of IBD.

Nanomaterials are defined as materials possessing at least one dimension within the range of 1–100 nm in three-dimensional space. The advancement of nanomaterials originated from an in-depth exploration of the microscopic realm. As materials transition to the nanoscale, they demonstrate unique physical and chemical properties that differ significantly from those of their macroscopic counterparts, thereby stimulating extensive research in the field (Demirel et al., 2015). X-ray spectroscopy can trace and characterize the chemical properties of nanomaterials by calibrating instruments, such as using radiation calibration for energy and wavelength-dispersive X-ray spectrometers. Combined with atomic X-ray fundamental parameters determination, it enables elemental analysis, morphological studies, and coordination research on nanomaterials (Beckhoff, 2022). Microscopy techniques like Atomic Force Microscopy (AFM), Transmission Electron Microscopy (TEM), and Scanning Electron Microscopy (SEM) are used for morphological characterization of nanomaterials (Aziz et al., 2025). Spectroscopic techniques, including fluorescence spectroscopy, solid-state nuclear magnetic resonance (NMR), infrared spectroscopy, and Raman spectroscopy, facilitate the molecular-level analysis of nanocomposites. These methodologies yield valuable insights into the surface properties of fillers, their dispersion states, and the interactions within the composite material (Bokobza, 2018). The nanoscale size range imparts unique physical, chemical, and biological properties to the materials, enabling them to exhibit superior performance across various fields compared to conventional materials (Youshia and Lamprecht, 2016) (Figure 2). The nanoscale is a primary characteristic for interaction with biological systems, as it determines the ability to penetrate cellular membranes, thereby facilitating traversal of biological barriers, interaction with the immune system, uptake, absorption, distribution, and metabolism (Banerjee et al., 2016). The surface chemistry of nanomaterials dictates their first interaction with tissues and cells, with surface charge being one of the primary aspects, along with their hydrophobic or hydrophilic properties (Wang et al., 2017). In medical applications, surface charge can be utilized to enhance the proximity of nanomaterials to epithelia, increasing their absorption and determining their specific interactions with intestinal epithelia (Bhalla et al., 2017). For instance, positively charged nanomaterials exhibit strong affinity for healthy epithelia, while negatively charged particles preferentially adhere to inflamed mucosa (Bandi et al., 2020). Another critical feature of nanomaterials is porosity, which quantifies the amount of void space within the material. Porous materials with numerous nanoscale pores allow for drug incorporation and retention, regulating their release to achieve controlled and sustained drug delivery systems (Trendafilova I et al., 2016). Interestingly, the impairment of the intestinal epithelial barrier caused by the aggregation of inflammatory substances paradoxically increases the likelihood of nanoparticle uptake by intestinal and immune cells (Xiao et al., 2016). The integration of nanotechnology and biomedicine has brought about groundbreaking advances in the treatment of IBD (Jacob et al., 2020). Nanotechnology facilitates the integration of small-molecule pharmaceuticals with organic or inorganic nanomaterials to create nanoscale drug formulations, referred to as nanomedicines. In contrast to conventional pharmaceuticals, nanomedicines are distinguished by their diminutive size, extensive surface area, and superior biocompatibility. These attributes significantly improve drug solubility, stability, targeting efficiency, absorption, and bioavailability (Wang et al., 2020).

## 4 The fundamental mechanisms of nanomaterials with aromatic functional groups in anti-inflammatory therapy

### 4.1 Targeted drug delivery

During the pathogenesis of inflammatory bowel disease, notable pathological alterations are observed in the blood vessels at sites of inflammation. The disruption of tight junction proteins (TJPs) and gap junctions within vascular endothelial cells results in the formation of large intercellular gaps, thereby significantly enhancing vascular permeability (Bhat et al., 2019). Nanomaterials, with their unique physicochemical properties and size characteristics within the 1–100 nm range, exhibit significant advantages in targeted accumulation at inflammatory sites (Raj et al., 2018). When nanomaterials circulate through the bloodstream and reach inflammatory regions, they exhibit unique pharmacokinetic behaviors. In comparison to small molecules, nanomaterials possess an extended serum half-life, enabling prolonged circulation. Furthermore, unlike large molecules, nanomaterials can more readily penetrate the highly permeable blood vessels and accumulate in inflamed tissues, owing to their distinct size and properties (Zhang et al., 2015; Cui et al., 2021). Furthermore, impaired lymphatic drainage at the inflammatory sites leads to fluid stasis and obstruction of pathways critical for maintaining fluid balance and material metabolism (Moulari et al., 2014). Studies have shown that this targeting capability is closely associated with the pathological features of the inflammatory sites, such as increased vascular permeability and impaired lymphatic drainage (Aibani et al., 2021). The clearance of nanomaterials accumulated in inflamed tissues via the lymphatic system is impeded, resulting in an extended retention time. This prolonged retention facilitates the targeted accumulation of nanomaterials in areas affected by IBD (Ali et al., 2016; Ana Melero et al., 2017).

### 4.2 Modulate immune response

Immune factors play a central role in the pathogenesis of IBD. Under typical conditions, the intestinal immune system sustains immune tolerance towards commensal microorganisms while preserving its capacity to mount a defense against pathogenic agents (Mann and Li, 2014). In patients with inflammatory bowel disease (IBD), the intestinal immune system is characterized by hyperactivation. Specifically, innate immune cells, including macrophages and dendritic cells, demonstrate atypical functions in the recognition and response to gut microbiota (Koelink et al., 2020). These cells release large amounts of pro-inflammatory cytokines, such as tumor necrosis factor-alpha (TNF-α) and interleukin-6 (IL-6), which trigger an inflammatory cascade. On the other hand, adaptive immune cells, such as T lymphocytes, display dysregulated activity. The hyperactivation of T helper1 (Th1) and T helper17 (Th17) cells leads to the secretion of numerous inflammatory mediators (Guan and Zhang, 2017; Sun et al., 2020). Furthermore, the insufficient number and reduced function of regulatory T cells (Tregs) impair their ability to effectively suppress excessive immune responses, resulting in persistent inflammation of the intestinal mucosa (Imamura et al., 2018). Nanomaterials with Aromatic Functional Groups can interact with surface receptors on macrophages, activating specific signaling pathways that drive the conversion of M1 macrophages to M2 macrophages (Na et al., 2019). Furthermore, they can influence the metabolic state of macrophages to regulate their polarization. Studies have shown that nanomaterials with antioxidant properties can reduce oxidative stress in macrophages, inhibit the activity of key glycolytic enzymes like pyruvate kinase M2 (PKM2), and promote M2 polarization, leading to decreased production of pro-inflammatory factors (Regmi et al., 2019). In animal experiments, mice with colitis treated with such nanomaterials exhibited an increased proportion of M2 macrophages in the gut, resulting in alleviated inflammatory symptoms (Mohammadi et al., 2019).

### 4.3 Antioxidant effect

It is well-known that reactive oxygen species (ROS) and reactive nitrogen species (RNS) are significantly produced at the sites of colonic inflammation (Dang et al., 2020). The ROS and RNS, in conjunction with oxidative stress and redox regulation, has been shown to be pivotal in the pathophysiology of both experimental animal models and patients with IBD. ROS and RNS encompass hydroxyl radicals (OH), superoxide anions, nitric oxide (NO), and peroxynitrite (OONO^−^). Persistently elevated levels of NO have been associated with severe chronic inflammatory conditions, including IBD, sepsis, rheumatoid arthritis, and systemic lupus erythematosus (Stettner et al., 2018). Carbon nanotubes (CNTs), characterized by their distinctive tubular structures and substantial specific surface areas, are capable of interacting with reactive oxygen species (ROS) such as superoxide anions (O_2_^−^). Specifically, the carbon atoms on the CNT surfaces can donate electrons to reduce O_2_^−^ to oxygen and water, thereby alleviating oxidative damage to cells. Similarly, fullerenes, with their cage-like configurations, have the ability to trap ROS.

### 4.4 Modulate gut microbiota

A substantial body of research has demonstrated a significant association between inflammatory bowel disease (IBD) and gut microbiota dysbiosis (Muhammad Shamoon and O’Brien, 2019; Ryan et al., 2020). Notably, this dysbiosis is characterized by a decreased prevalence of beneficial bacterial genera, including Bifidobacterium and *Lactobacillus*, alongside an increased relative abundance of pathogenic bacteria, such as *Escherichia coli* and members of the Enterobacteriaceae family (Hofer, 2014; Franzosa et al., 2019). This microbial imbalance can disrupt the function of the intestinal mucosal barrier, increase intestinal permeability, and facilitate the penetration of bacteria and their products into the submucosa of the intestine (Johansson et al., 2014; Rodriguez-Pineiro and Johansson, 2015). This, in turn, activates the immune system and triggers an inflammatory response (Hofer, 2014). Therefore, the use of nanomaterials to regulate the gut microbiota represents a promising therapeutic strategy for the treatment of IBD (Xiu Wang et al., 2024). Liu et al. designed colon-targeting adhesive hydrogel microspheres that degrade in the colon environment, releasing specific factors to optimize the composition of the gut microbiota (Liu et al., 2021).

## 5 Common nanomaterials containing aromatic functional groups

Nanomaterials incorporating aromatic functional groups are prevalent in both natural plant sources and synthetic compounds. These materials can be systematically classified into natural polyphenols, flavonoids, and other categories based on their distinct chemical structures and functional characteristics. The natural polyphenol category includes compounds such as curcumin and proanthocyanidins, whereas the flavonoid category comprises substances like baicalein and quercetin.

### 5.1 Curcumin

**FIGURE 3:**
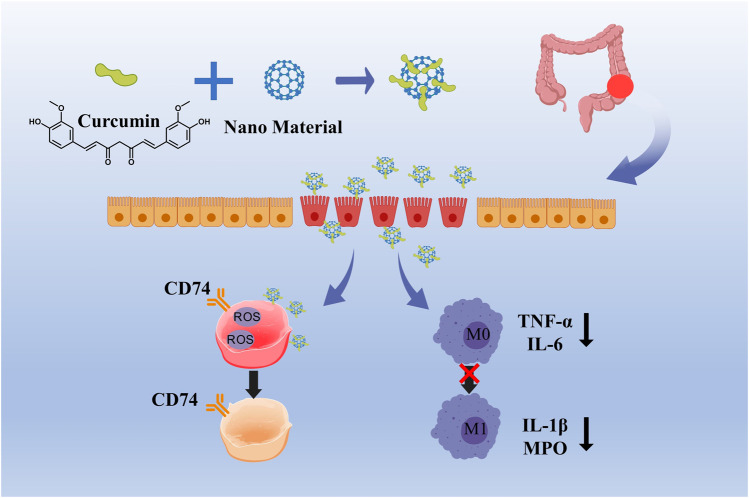
Curcumin relieves oxidative damage by scavenging ROS and inhibiting the transformation of M1 macrophages into M2 macrophages.

Curcumin, a bioactive compound derived from turmeric (Curcuma longa), has garnered considerable interest in the domain of nanomedicine for the treatment of inflammatory bowel disease (IBD), owing to its distinctive aromatic functional groups and extensive range of biological activities (Beloqui et al., 2016; Karthikeyan et al., 2021). The molecular structure of curcumin is characterized by two benzene rings linked via an α,β-unsaturated carbonyl group, and includes various bioactive functional groups such as hydroxyl (-OH), methoxy (-OCH_3_), and an α,β-unsaturated ketone (C=O) group. The presence of conjugated double bonds within the benzene rings enhances curcumin’s antioxidant capabilities, facilitating the scavenging of free radicals and the inhibition of ROS accumulation, which in turn mitigates oxidative damage to intestinal tissues (Guo et al., 2022). Curcumin exerts its anti-inflammatory effects through multiple mechanisms, including the regulation of M1/M2 macrophage polarization, inhibition of the TLR signaling pathway, suppression of NLRP3 inflammasome activation, modulation of the Treg/Th17 cell balance, and downregulation of NF-κB signaling pathway components, thereby inhibiting the release of pro-inflammatory cytokines and reducing oxidative stress levels (Liu et al., 2013; Hasanzadeh et al., 2020; Laurindo et al., 2023) (Figure 3). Despite its great pharmacological activities, curcumin is highly hydrophobic, which results in poor bioavailability. A simple way of solving the limiting factors of curcumin is to improve its bioavailability, protect it from degradation and meta bolism (Khezri et al., 2021). Various types of nanoparticles (NPs), such as polymer NPs, poly meric micelles, liposome/phospholipid, nano-/microemulsions, nanogels, solid lipid NPs, polymer conjugates, self-assemblies, and so on, are suitable for the delivery of an active form of curcumin (Hegde et al., 2023).

Pharmacokinetic analysis following oral administration of nano-curcumin in murine models revealed an approximately 20-fold decrease in the required dosage compared to unformulated curcumin to attain similar concentrations in plasma and central nervous system (CNS) tissues (Szymusiak et al., 2016). Furthermore, the aromatic ring structure of curcumin facilitates its ability to chelate metal ions, which can be exploited to develop metal-polyphenol network nanocarriers, presenting potential strategies for improved drug targeting (Coelho et al., 2020).

### 5.2 Proanthocyanidins

**FIGURE 4:**
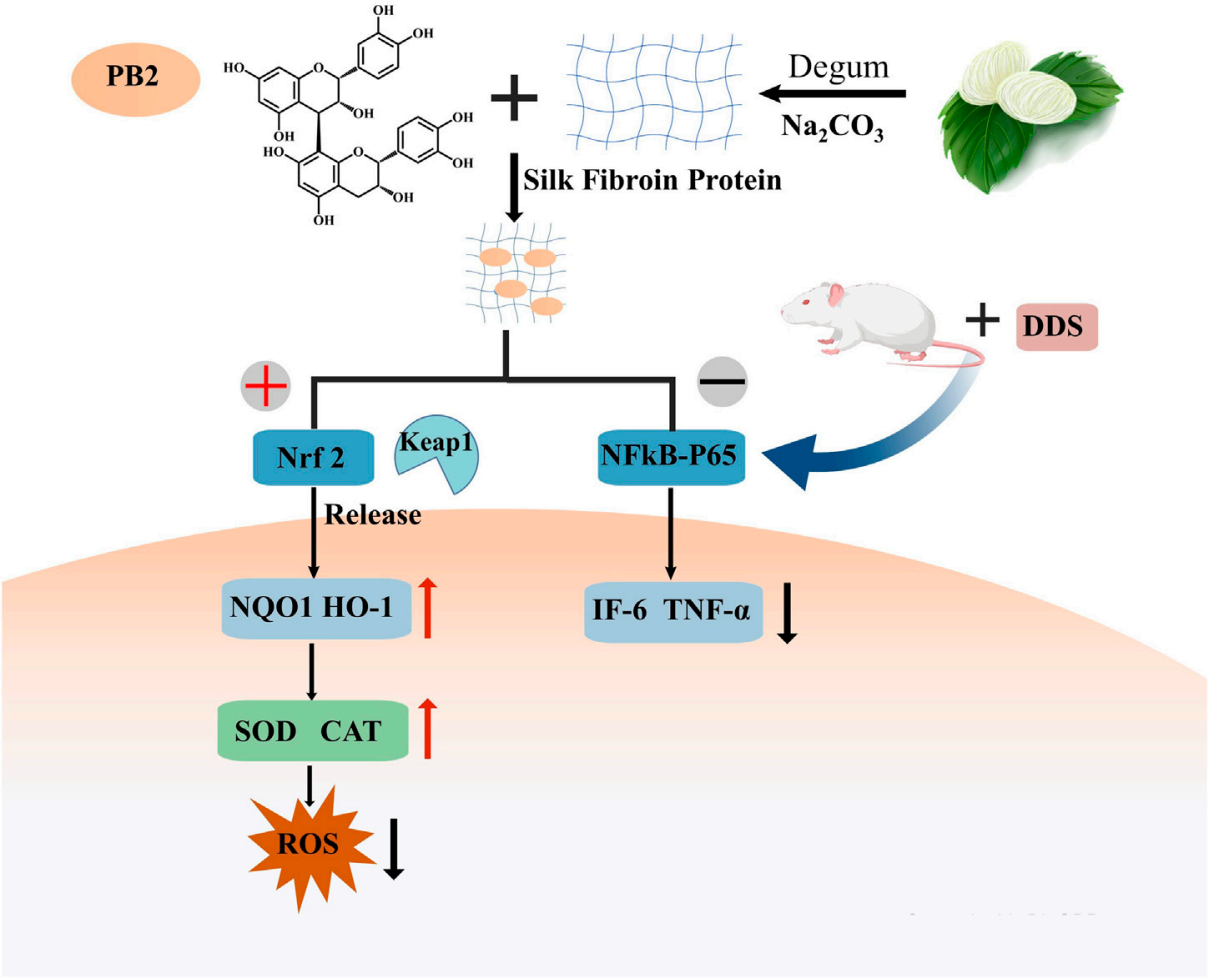
Procyanidin B2 alleviated the symptoms of DDS-induced ulcerative colitis by activating the Nrf2 signaling pathway and inhibiting the nuclear localization of p65.

Proanthocyanidins (PACs) constitute a class of polyphenolic compounds that are prevalent in various plant species. These compounds have been extensively characterized for their diverse biological and pharmacological properties, which encompass antioxidant, antibacterial, antiviral, anti-inflammatory, and anti-allergic activities. The molecular structure of PACs is distinguished by a conjugated system of multiple aromatic rings and phenolic hydroxyl groups, endowing them with potent antioxidant capabilities. In the context of IBD treatment, the antioxidant properties of PACs are of particular importance. Empirical studies have demonstrated that PACs are capable of scavenging ROS, mitigating oxidative stress-induced damage, and inhibiting the release of inflammatory mediators (Wang et al., 2022b). Proanthocyanidins, characterized by multiple phenolic hydroxyl groups within their molecular structure, exhibit heightened susceptibility to environmental factors including light, pH, and redox conditions (Luo et al., 2020). Their absorption predominantly occurs in the small intestine; however, this process is impeded by their substantial molecular weight and degree of polymerization (Tao et al., 2019). These characteristics contribute to the inherent instability and limited permeability of proanthocyanidins, thereby restricting the quantity of these compounds that enter the systemic circulation in their native or active form (Fernandez et al., 2016). To put it briefly, the application of proanthocyanidins is limited by their chemical instability and low bioavailability, even though they have desirable properties. To tackle the challenges of PC’s low oral bioavailability and instability, encapsulation methods have been adopted as a successful strategy to improve its shortcomings. A study explored the creation of proanthocyanidin-loaded chitosan nanoparticles (PC-CS-NPs) using ionotropic gelation. These nanoparticles, characterized by FTIR, XRD, and DLS, were under 300 nm in size, spherical, smooth, and uniform, as shown by SEM. *In vitro* tests revealed a sustained release of proanthocyanidins in different buffers. Additionally, PC-CS-NPs showed equal or superior effectiveness in scavenging DPPH and ABTS free radicals compared to native drugs (Ding et al., 2021). Sericin (SS), a biologically active polymer, demonstrates exceptional biocompatibility and bioactivity, indicating significant potential for drug delivery applications. Researchers have integrated proanthocyanidins (PACs) into sericin to develop SS/PAC composite materials, which synergize high antioxidant capacity with superior biocompatibility. The SS/PAC composites exhibit uniform dispersion in aqueous solutions, with an average particle diameter of approximately 136 nm, and achieve a substantial drug loading capacity of 1767 mg/g (Wang et al., 2022a). The SS/PAC demonstrated strong antioxidant properties and outstanding biocompatibility in both laboratory and living organism settings, and it could ease DSS-induced ulcerative colitis symptoms by managing oxidative stress, reducing inflammation, and repairing tissue damage. This strategy of combining proanthocyanidins with nano-carriers successfully addresses the potential stability and targeting challenges faced by PACs in biomedical applications, significantly enhancing their therapeutic efficacy (Figure 4).

### 5.3 Quercetin

Quercetin is a flavonoid compound extracted from plants, exhibiting excellent anti-inflammatory and antioxidant activities (Cui et al., 2022). Quercetin is a yellow, crystalline plant polyphenolic flavonoid, distinguished by its chemical composition of five hydroxyl groups and three benzene rings (Batiha et al., 2020; Aghababaei and Hadidi, 2023). This distinctive molecular structure underpins its wide range of biological activities and potential therapeutic applications. Quercetin exhibits numerous therapeutic effects, including antioxidant, anti-inflammatory, anti-apoptotic, and anti-aging properties, which are mediated through intricate mechanisms involving the modulation of various signaling pathways (Chen et al., 2018; Cui et al., 2022). In the realm of oncological therapeutics, quercetin is predominantly employed as an adjuvant treatment. Its co-administration with natural compounds, including curcumin, resveratrol, and green tea polyphenols, has been shown to markedly potentiate their anti-tumor efficacy (Joshi et al., 2023). Furthermore, quercetin enhances the sensitivity of cancer cells to chemotherapeutic agents and radiotherapy. For example, when combined with doxorubicin and cisplatin, quercetin allows for a reduction in the required dosage of these drugs, thereby minimizing side effects while preserving therapeutic efficacy (Xu et al., 2021). Notably, a mouse study has shown that quercetin also has the potential to alleviate inflammation (Schwingel et al., 2014; Dong et al., 2020). Quercetin’s anti-inflammatory effects are mediated through multiple mechanisms. It suppresses the activation of Toll-like receptor 4 (TLR4), thereby reducing the expression of inflammatory mediators and cytokines (Chen et al., 2018). Quercetin also inhibits the overexpression of adhesion molecules and chemokines, preventing inflammation. Additionally, it regulates immune responses by enhancing interferon-γ (IFN-γ) production in Th1 cells while reducing IL-4 levels in Th2 cells. Furthermore, quercetin reduces the levels of key inflammatory molecules, including COX-2, NF-κB, MAPK, and CRP, contributing to its anti-inflammatory activity (Haddad, 2002; Li et al., 2016).

Despite its promising therapeutic potential, quercetin’s poor solubility and low bioavailability have limited its clinical application (Moradi et al., 2020; Tomou et al., 2023; Zhang et al., 2023). To address this challenge, researchers have developed various nanosystems, such as nanoemulsions, liposomes, lipid nanoparticles, nanostructured lipid carriers (NLC), solid lipid nanoparticles (SLN), and mesoporous silica, to enhance its bioavailability and therapeutic efficacy (Chessa et al., 2011; Bose et al., 2013; Scalia et al., 2013; Sapino et al., 2015; Abel Lozano‐Perez et al., 2017). These systems are designed to enhance the absorption and distribution of quercetin, thereby optimizing its therapeutic efficacy. Beyond these nanosystems, researchers have engineered a vesicular delivery system encapsulated with chitosan/lecithin and loaded with quercetin. This system provides benefits in terms of stability, controlled release, and targeted delivery, further augmenting the therapeutic performance of quercetin (Kumari et al., 2010; Castangia et al., 2015).

### 5.4 Baicalein

Baicalein (3,3′,4′,5,6-pentahydroxyflavone) is a highly effective antioxidant prevalent in numerous plant species, with a notable concentration in the roots of Scutellaria baicalensis. It demonstrates a broad spectrum of physiological activities, encompassing neuroprotective, antioxidant, antibacterial, antiviral, antiallergic, anti-inflammatory, and antitumor properties (Gao et al., 2016; Wei et al., 2017). A study employing a murine model revealed that baicalein exhibits anti-inflammatory properties in macrophages stimulated by double-stranded RNA (dsRNA). This effect is mediated through the suppression of nitric oxide (NO), cytokines, chemokines, and growth factors production via the endoplasmic reticulum stress-CHOP/STAT signaling pathway (Raghavendra et al., 2016). Baicalein’s structure includes three adjacent phenolic hydroxyl groups, which facilitate the formation of intramolecular hydrogen bonds. This structural feature contributes to its low hydrophilicity and water solubility, ultimately resulting in poor oral bioavailability (Li et al., 2018; Pi et al., 2019). To address these limitations, various strategies have been reported to enhance the solubility, stability, and bioavailability of baicalein. The use of nano-liposomes is an effective method (Liu et al., 2016).

A notable advancement in this field involves the development of a highly efficient method for preparing glycyrrhizic acid–baicalein (GA-BE) nano-micelles. The nano-micelles produced using the optimal formulation were characterized via differential scanning calorimetry (DSC) and Fourier-transform infrared spectroscopy (FT-IR), confirming that baicalein existed in a non-crystalline state within the micelles and that a conjugated aromatic system was present. This formulation increased the water solubility of baicalein by a factor of 4,600. *In vitro* drug release studies demonstrated that the nano-micelles exhibited a sustained-release effect, which could be partially controlled by adjusting the pH (You et al., 2021).

## 6 Challenges and future directions

Nanomaterials have demonstrated potential advantages in the treatment of IBD, including enhanced efficacy and targeted accumulation in affected tissues. However, the transition from laboratory research to clinical application presents substantial challenges. The foremost obstacle is the absence of regulatory approval for clinical use, as no nanomaterials have yet been sanctioned for IBD treatment. This underscores the significant barriers involved in translating laboratory findings into clinical practice. To address this, there is an urgent need for large-scale, multi-center, rigorously controlled clinical trials to thoroughly assess the safety and efficacy of nanomaterials in accordance with regulatory standards. These trials should encompass long-term efficacy monitoring and surveillance for adverse reactions. Furthermore, the side effects and toxicity of nanomaterials in human cells remain insufficiently explored. Due to their nanoscale dimensions, nanomaterials interact with biomolecules and cells in ways that differ from conventional drugs, complicating precise toxicity risk assessments. This represents a significant challenge to their clinical application. Therefore, establishing a systematic nanomaterial toxicity assessment framework is imperative. This framework should encompass the integration of *in vitro* cell experiments, *in vivo* animal models, and advanced omics technologies to comprehensively examine the interaction mechanisms between nanomaterials and cells or biomolecules, as well as to predict potential toxicological effects. The translation of research findings from animal models to humans presents significant challenges. The physiological structures, immune systems, and disease pathologies of different animal models differ from those of humans, rendering direct extrapolation of therapeutic effects and safety profiles observed in animal models to humans unfeasible. For instance, certain nanomaterials may demonstrate promising therapeutic effects in murine models, but variations in pharmacokinetics or biodistribution in larger animals or humans may result in diminished efficacy or unforeseen toxic reactions. Furthermore, the experimental conditions of animal models differ from those of human clinical environments, which further complicates the translation process. To address this issue, it is essential to strengthen the connection between preclinical and clinical research by applying the principles and methods of translational medicine. Incorporating more clinically relevant indicators and parameters into preclinical studies, conducting dose-escalation studies, and initiating early clinical trials can help gradually verify the safety and efficacy of nanomaterials in humans.

Intelligent responsive nanosystems present substantial potential for the treatment of IBD by facilitating precise drug delivery and personalized therapeutic interventions, which could markedly improve therapeutic outcomes and enhance patients’ quality of life. Nonetheless, these systems remain in the research phase, with challenges such as ensuring biological safety and achieving large-scale production needing resolution to facilitate their clinical translation and application. The implementation of personalized therapy is anticipated to hold critical significance in future clinical practice. In designing personalized nanotherapeutic delivery systems, gene biomarkers can be utilized for targeted delivery. By identifying specific biomarkers (e.g., particular proteins or nucleic acids) associated with IBD based on patients’ genetic characteristics, nanotherapeutic delivery systems can be designed with surface modifications that recognize the ligands of these biomarkers, enabling precise targeting and drug delivery to the affected sites.
